# ‘Acute myeloid leukemia: a comprehensive review and 2016 update'

**DOI:** 10.1038/bcj.2016.50

**Published:** 2016-07-01

**Authors:** I De Kouchkovsky, M Abdul-Hay

**Affiliations:** 1 Department of Medicine, New York University School of Medicine, New York, NY, USA; 2 Department of Hematology/Oncology, New York University Perlmutter Cancer Center, New York, NY, USA

## Abstract

Acute myeloid leukemia (AML) is the most common acute leukemia in adults, with an incidence of over 20 000 cases per year in the United States alone. Large chromosomal translocations as well as mutations in the genes involved in hematopoietic proliferation and differentiation result in the accumulation of poorly differentiated myeloid cells. AML is a highly heterogeneous disease; although cases can be stratified into favorable, intermediate and adverse-risk groups based on their cytogenetic profile, prognosis within these categories varies widely. The identification of recurrent genetic mutations, such as FLT3-ITD, NMP1 and CEBPA, has helped refine individual prognosis and guide management. Despite advances in supportive care, the backbone of therapy remains a combination of cytarabine- and anthracycline-based regimens with allogeneic stem cell transplantation for eligible candidates. Elderly patients are often unable to tolerate such regimens, and carry a particularly poor prognosis. Here, we review the major recent advances in the treatment of AML.

## Introduction

Acute myeloid leukemia (AML) is the most common acute leukemia in adults, accounting for ~80 percent of cases in this group.^1^ Within the United States, the incidence of AML ranges from three to five cases per 100 000 population. In 2015 alone, an estimated 20 830 new cases were diagnosed, and over 10 000 patients died from this disease.^2^ The incidence of AML increases with age, from ~1.3 per 100 000 population in patients less than 65 years old, to 12.2 cases per 100 000 population in those over 65 years. Although advances in the treatment of AML have led to significant improvements in outcomes for younger patients, prognosis in the elderly who account for the majority of new cases remains poor.^3^ Even with current treatments, as much as 70% of patients 65 years or older will die of their disease within 1 year of diagnosis.^4^

## Pathophysiology

AML can arise in patients with an underlying hematological disorder, or as a consequence of prior therapy (for example, exposure to topoisomerases II, alkylating agents or radiation).^5^ However in majority of cases, it appears as a *de novo* malignancy in previously healthy individuals. Regardless of its etiology, the pathogenesis of AML involves the abnormal proliferation and differentiation of a clonal population of myeloid stem cells. Well-characterized chromosomal translocations, such as t(8:21) in core-binding factor AML (CBF-AML) or t(15:17) in acute promyelocytic leukemia (APL) result in the formation of chimeric proteins (RUNX1-RUNX1T1 and PML-RARA, respectively), which alter the normal maturation process of myeloid precursor cells. In addition to large chromosomal rearrangements, molecular changes have also been implicated in the development of AML. In fact, genetic mutations are identified in more than 97% of cases,^6^ often in the absence of any large chromosomal abnormality.^7^ Studies of animal models at the turn of the century led to the development of a two-hit model of leukemogenesis, which offers a conceptual framework for classifying the various mutations associated with AML.^8^ According to this model, class I mutations which result in the activation of pro-proliferative pathways must occur in conjunction with class II mutations which impair normal hematopoietic differentiation in order for leukemia to develop.^9, 10^ Common class I mutations, such as FLT3 (internal tandem duplications, ITD, and tyrosine kinase domain mutations, TKD), K/NRAS, TP53 and c-KIT are found in ~28, 12, 8 and 4% of cases, respectively.^7^ Studies of solid and hematological malignancies have also highlighted the role of signal transducer and activator of transcription 3 (STAT3) in the stimulation of cellular proliferation and survival.^11, 12, 13^ Enhanced tyrosine phosphorylation of STAT3 whether due to increased secretion of cytokines, such as IL-6(ref. 14) or mutations in receptor tyrosine kinases (for example, FLT3 duplications^15^ or less frequently JAK2)^16^ is seen in up to 50% of AML cases and signifies a worse prognosis. Notable class II mutations include NPM1 and CEBPA, which are found in ~27% and 6% of cases, respectively, and confer a better prognosis.^7^ Alterations in genes involved in epigenetic regulation have recently emerged as a third class of mutations, with downstream effects on both cellular differentiation and proliferation. These include mutations in the DNA-methylation related genes DNMT3A, TET2, and IDH-1 and IDH-2,^6, 7^ which are found in more than 40% of AML cases.

Despite significant advances, much remains to be discovered on the exact contribution of these individual mutations to the development of AML. As suggested by the ‘two-hit model,' the pathogenesis and behavior of AML depends heavily on the interactions between different somatic alterations and chromosomal rearrangements. Thus, the c-KIT mutation has been associated with t(8;21) or inv(16), and its presence carries significant implications regarding prognosis. Similarly, NMP1 (a class II mutation) frequently occurs in conjunction with the class I mutation FLT3-ITD, or mutations in the epigenetic genes DNMT3A and IDH-1 or IDH-2.^6^

Most of the clinical manifestations of AML reflect the accumulation of malignant, poorly differentiated myeloid cells within the bone marrow, peripheral blood and infrequently in other organs. The majority of patients presents with a combination of leukocytosis and signs of bone marrow failure such as anemia and thrombocytopenia. Fatigue, anorexia and weight loss are common complaints; lymphadenopathy and organomegaly are not typically seen. If left untreated, death usually ensues within months of diagnosis secondary to infection or bleeding. The diagnosis of acute leukemia is established by the presence of 20% or more blasts in the bone marrow or peripheral blood.^17^ AML is further diagnosed by demonstrating the myeloid origin of these cells through testing for myeloperoxidase activity, immunophenotyping or documenting the presence of Auer rods. The latter finding consists of azurophilic, often needle-shaped cytoplasmic inclusion bodies that are commonly seen in APL, acute myelomonocytic leukemia and the majority of AML with t(8;21). The diagnosis of AML can also be established in the presence of an extramedullary tissue infiltrate, or a documented t(8;21), inv(16) or t(15;17) in the appropriate clinical setting, regardless of the blast percentage.^18^

## Classification

The French–American–British classification system represents the first attempt to distinguish between different types of AML. Established in 1976, it defines eight subtypes (M0 through M7) based on the morphological and cyto-chemical characteristics of the leukemic cells. In 2001, as part of an effort to integrate advances made in the diagnosis and management of AML, the World Health Organization (WHO) introduced a new classification system followed by a revised version in 2008.^18^ Later in 2016 a new revised version was released, the WHO classification of AML distinguishes itself by incorporating genetic information with morphology, immunophenotype and clinical presentation to define six major disease entities: AML with recurrent genetic abnormalities; AML with myelodysplasia-related features; therapy-related AML; AML not otherwise specified; myeloid sarcoma; and myeloid proliferation related to Down syndrome (Table 1).^19^ Among cases of AML with recurrent genetic abnormalities, 11 subtypes are further delineated according to distinct chromosomal translocations. In addition, the provisional entities AML with mutated NPM1 and AML with mutated CEBPA were introduced as part of the 2008 revision,^18^ while AML with BCR-ABL1 and AML with mutated RUNX1 were introduced as part of the 2016 revision.^19^ Genetic abnormalities also inform the diagnosis of AML with myelodysplasia-related changes: along with a history of MDS or morphological evidence of dysplasia in two or more myeloid cell lineages, the presence of myelodysplasia-related cytogenetic abnormalities such as monosomy 5 or 7, and deletion 5q or 7q identify cases of AML with myelodysplasia-related features.

## Prognostic factors

Accurate assessment of prognosis is central to the management of AML. By stratifying patients according to their risk of treatment resistance or treatment-related mortality (TRM), prognostic factors help guide the physician in deciding between standard or increased treatment intensity, consolidation chemotherapy or allogenic hematopoietic stem cell transplant, or more fundamentally in choosing between established or investigational therapies. Among clinical factors, increased age and poor performance status are both associated with lower rates of complete remission (CR) and decreased overall survival (OS).^3, 20^ Age and performance status at diagnosis similarly help to predict the risk of TRM, although multivariate model analyses suggest that other variables such as platelet count, serum creatinine or albumin rather than age itself account for most of the increased risk of TRM seen in older patients.^21^ Therapy-related AML and AML associated with a prior hematological malignancy also carry a significantly poorer prognosis.^22^ Although clinical factors have an important role in guiding therapy, cytogenetic changes constitute the single strongest prognostic factor for CR and OS in AML. Accordingly, cases of AML can be stratified into favorable, intermediate or adverse prognostic risk groups based on their cytogenetic profile alone. The chromosomal rearrangements t(8;21), t(15;17) or inv(16) all confer a favorable prognosis,^17, 23^ with a 3 year OS of 66% and 33% in patients younger and older than 60 years, respectively.^24^ In contrast, cytogenetic changes such as a complex karyotype (that is, three or more chromosomal abnormalities in the absence of any of the recurrent genetic abnormalities identified in the WHO 2008 classification), monosomy 5 or 7, t(6;9), inv(3) or 11q changes other than t(9;11) have all been associated with a significantly higher risk of treatment failure and death (Table 2).^24^ AML cases with an intermediate prognostic risk mainly constitute of patients with normal cytogenetics (CN-AML).^17, 24^

Gene mutations have helped further refine risk stratification based on cytogenetic changes alone. Among patients with t(8;21), the presence of a c-KIT mutation significantly increases the risk of relapse, and decreases OS to levels comparable to those of patient with intermediate-risk AML.^6, 23, 25^ Although there is some evidence that the presence of c-KIT mutations similarly lowers prognosis in patients with inv(16),^26^ recent studies have failed to show any prognostic impact in this subset of cases.^6, 27, 28^ Molecular changes have a particularly important role in refining the prognosis of patients with CN-AML, which includes nearly half of *de novo* AML cases. Thus CN-AML with a mutated CEBPA or a mutated NPM1 in the absence of FLT3-ITD has been identified as having a prognostic risk similar to that of AML with favorable cytogenetic changes.^17, 29^ The favorable prognostic impact of CEBPA mutations has been further refined to biallelic mutations only.^30^ On the other hand, multiple studies^29, 31^ including a meta-analysis of relapse-free survival (RFS) and OS in patients with CN-AML <60 years of age have consistently shown the presence of FLT3-ITD to be associated with a worsened prognosis.^6, 32^ This has led to the classification of CN-AML with FLT3-ITD into the adverse prognostic-risk group.^23, 33^ As with CEBPA mutations, the prognostic impact of FLT3-ITD may depend on the presence of biallelic mutations. Several studies have shown a significantly worse prognosis in patients with higher mutant to wild-type allelic ratios.^34, 35^ TP53 mutations, which are found in only 2– 8% of cases,^6, 7^ occur more frequently in cases with unfavorable cytogenetic and a complex karyotype.^36^ However regardless of the cytogenetic profile, TP53 mutations are associated with a very poor prognosis^10^ and may in fact represent the single worst genetic prognostic factor.^36^ Mutations in DNA-related genes also carry important implications for the prognosis and treatment of AML. The presence of a mutated DNA methyl transferase gene *DNMT3A* has been associated with a worsened prognosis in CN-AML and adverse-risk AML.^37^ Partial tandem duplications of KMT2A (previously known as MLL), which encodes a histone methyltransferase, have also been associated with a worse prognosis in CN-AML.^6, 10, 36^ The prognostic impact of IDH-1/IDH-2 mutations is less well established and is likely modified by co-occurring mutations. Among cases of FLT3-ITD-negative and NPM1-mutated CN-AML, IDH-1/IDH-2 mutations have been shown to improve OS.^6^ However a recent study of 826 patients with known IDH-1 and IDH-2 status found no prognostic impact on treatment response or OS.^38^ Further analysis is required to delineate the role of DNA-related genes in OS and treatment response. In addition to genetic profiling at the time of diagnosis, information gained after treatment initiation plays a growing role in refining patient prognosis: As could be expected, individuals who achieve CR (defined morphologically as a blast count of <5% of total nonerythroid cells in the bone marrow) after induction therapy have a significantly increased survival compared with patients with treatment resistant AML.^39, 40^ Survival among patients achieving CR is further influenced by the correction or persistence of thrombocytopenia, with a shorter duration of survival observed in the latter group.^40^ More recently, techniques such as real-time PCR and flow cytometry have been used to measure the presence of minimal residual disease among patients in CR. Persistently elevated levels of RUNX1-RUNX1T1 transcripts after induction therapy in patients with t(8;21) AML are thus associated with an increased incidence of relapse.^41, 42^ Similarly among patients with intermediate-risk disease, detection of minimal residual disease by flow cytometry is an independent predictor of relapse and survival^43, 44^ and carries important implications for management.^45^

## Established treatments

Eligible patients first undergo induction therapy to achieve CR. Unfortunately, minimal residual disease often persists in CR, and relapse will inevitably occur if treatment is discontinued. Therefore, a favorable response to induction therapy should be followed by consolidation therapy in order to eradicate any residual disease and achieve lasting remission. The mainstay of induction therapy consists of the ‘7+3' regimen, which combines 7 days of continuous infusion cytarabine with 3 days of anthracycline. It is generally offered to patients with an intermediate to favorable prognosis and a low risk of TRM (for example, younger patients with good performance status, normal creatinine, albumin and platelet count).^23^ Studies of induction regimens using either daunorubicin at 60 or 90 mg/m^2^, or idarubicin at 12 mg/m^2^ have shown similar rates of CR and survival.^23, 46, 47^ A subset of patients with DNMT3A and KMT2A mutations, which represents a poor prognostic marker, may however benefit from higher doses of daunorubicin.^6^ Standard dosing of cytarabine consists of 100–200 mg/m^2^ daily administered as a continuous infusion over 7 days. Although studies have shown greater efficacy at higher doses, this added benefit is small and accrued at the cost of increased toxicity;^23, 48, 49^ induction therapy with high-dose cytarabine is generally reserved for refractory disease. The combination of fludarabine, cytarabine, G-CSF and idarubicin (FLAG-IDA), which was traditionally used for the treatment of relapse, has also been shown to be a reasonable alternative to standard induction regimens and results in similar CR rates and OS overall but higher rates of CR after a single course.^50^

An optimal approach to elderly patients with AML has not been established. Individuals over the age of 65 are more likely to present with an adverse cytogenetic-risk profile, are less likely to respond to chemotherapy and are often more susceptible to treatment-related toxicities. However despite a significantly worse prognosis, induction therapy improves survival in patients over the age of 65 when compared with supportive care and palliative chemotherapy,^51^ and should be pursued whenever possible. Hypomethylating agents, traditionally used for the treatment of myelodysplastic syndrome (MDS), have also shown efficacy in elderly patients with AML. Evidence of a therapeutic benefit was first demonstrated in *post hoc* analyses of patients with MDS who were retrospectively found to meet diagnostic criteria for AML under the WHO classification.^52^ A 2012 randomized trial of the hypomethylating agent decitabine versus supportive care or low-dose cytarabine in patients 65 years or older showed a significant improvement in OS with hypomethylating therapy (although this survival advantage failed to meet statistical significance in the primary analysis).^53^ A more recent trial comparing the hypomethylating agent azacitidine to supportive care, low-dose cytarabine or standard induction therapy in patients 65 years or older did not show any significant improvement in median OS.^54^ However among patients pre-selected to receive supportive care, a subgroup analysis suggested a benefit to azacitidine therapy. A similar benefit was seen among patients with adverse cytogenetic-risk profile or MDR-AML.^54^ These results suggest a promising role for the use of hypomethylating agents in older individuals, including as a bridge for induction chemotherapy with the goal of achieving CR.

Response to induction therapy should be evaluated 14 days after initiation of treatment with a bone marrow aspirate and core biopsy.^17^ Up to 25–50% of patients show persistent cytological evidence of disease after one cycle of standard induction therapy and require reinduction.^55^ Treatment options for such patients (as well as patients with a disease relapse) include a second cycle of standard dose cytarabine combined with an anthracycline, high-dose cytarabine alone or FLAG-IDA, with roughly similar CR rates of up to 50%.^23, 56, 57^ In addition, mitoxantrone-based regimens (in combination with etoposide and/or cytarabine) have been shown to achieve CR up to 40–60% of patients with recurrent or refractory AML.^58, 59^ Ultimately, around 60–80% of patients with *de novo* AML will achieve CR with induction therapy.^60^ Patients in remission should be offered consolidation therapy to eradicate residual disease and prevent relapse. Available options for consolidation include chemotherapy and allogeneic hematopoietic stem cell transplant (allo-HSCT). When choosing between these different options, the risk of TRM should be weighted against the risk of treatment failure or relapse. Intention-to-treat analyses (allocating patients to allo-HSCT or chemotherapy based on the availability of a related-donor) have found no benefit to allo-HSCT when compared with chemotherapy in patients with cytogenetically favorable AML in first CR.^61, 62^ Thus chemotherapy is a reasonable first-line consolidation choice for patients with a favorable prognosis. Regimens generally consist of intermediate-dose cytarabine (two to four cycles each consisting of six doses at 1.5–3 g/m^2^), which has been shown to be as effective as high-dose cytarabine^48, 50, 63^ or multi-agent regimens.^50^ The optimal choice of consolidation therapy for patients with an intermediate-risk cytogenetic profile but favorable genetic mutations is more controversial: several studies have found no benefit to transplantation in patients with NPM1-mutated, FLT3-ITD-negative CN-AML.^64, 65^ However in a recent intention-to-treat analysis, allo-HSCT was shown to improve RFS in this subset of patients.^66^ While these conflicting results may reflect differences in study design, the difference in observed outcomes may also reflect the modulating effect of co-occurring mutations, such as IDH-1/-2.^6^ On the other hand allo-HSCT significantly prolongs RFS and OS in some patients with intermediate-risk and in most with adverse-risk AML, and should be offered as a first-line consolidation therapy in eligible patients.^62, 67, 68, 69^ In addition, allo-HSCT has been shown to prolong RFS and improve OS in patients with CN-AML and a high FLT3-ITD allelic ratio.^70^

## Novel agents

### FLT3-ITD inhibitors

Inhibition of tyrosine kinase (TK) receptors has been used successfully in various solid and hematological malignancies, including Philadelphia-chromosome positive leukemias. Given the prognostic impact and the high rate of FLT3 mutations, inhibition of this TK has long been recognized as a potential therapeutic target in AML. Tested agents include the first-generation inhibitors sorafenib and midostaurin, as well as newer second-generation agents such as quizartinib and crenolanib.

#### Sorafenib

Sorafenib is a tyrosine kinase inhibitors (TKI) of RAF kinase, c-KIT, VGFR, PGFR and FLT3-ITD, which was first used for the treatment of hepatocellular and renal cell carcinoma. As early as 2008, phase I trials of sorafenib administered as a single agent n patients with FLT3-ITD-positive relapsed or refractory (r/r) AML demonstrated significant reductions in the number of leukemic cells both in the peripheral blood and bone marrow, achieving CR in several patients.^71, 72, 73, 74^ In a phase II trial of 13 patients with r/r FLT3-ITD-positive AML, single-agent sorafenib at doses of 200–400 mg twice daily established CR (including CR with insufficient hematologic recovery) in over 90% of cases. The agent was well-tolerated, with grades 3 to 4 adverse events consisting of hyperbilirubinemia (in 4/13 patients), elevated transaminases (5/13), diarrhea (4/13), rash (2/13), pancreatitis (1/13), colitis (1/13), pericarditis (1/13), hand and foot syndrome (2/13) and elevated creatinine (1/13). Yet despite a strong initial response, the majority of patients relapsed within 72 days of remission. Treatment failure was associated with the emergence of D835Y and D835H mutations within the FLT3 TKD. The addition of sorafenib to standard induction regimens has yielded similarly mixed results: although initial phases I and II trials of combination therapy were able to achieve longer periods of disease-free survival, relapse invariably ensued within several months of treatment.^75, 76, 77^ In one such study, the combination of sorafenib with cytarabine and idarubicin as induction and consolidation therapy was able to achieve CR (or CR with an incomplete platelet recovery) in 18 out of 18 patients with previously untreated FLT3-ITD-positive AML.^75^ However after a median follow-up of 9 months, more than half of these patients had relapsed. Interestingly, no new mutation was observed in the FLT3 TKD of the relapsed samples available for genetic sequencing. Alternative mechanisms of resistance, such as the increased levels of FLT3 receptor ligand seen in patients receiving standard chemotherapy, have been postulated to contribute to treatment failure.^78^ Combinations of sorafenib and hypomethylating agents, which have not been associated with an increase in FLT3 receptor ligand levels, are currently being investigated. An encouraging phase II trial of sorafenib and azacitidine in 43 patients with relapsed/refractory AML reported a response rate of 46%, including 16% CR and 27% CR with incomplete count recovery.^79^

The role of sorafenib as a first-line therapy in AML was further delineated in two recent randomized trials: In 2013, Serve *et al.* published the results of study, in which 201 patients 60 years or older with newly diagnosed AML were randomized to receive sorafenib or placebo in addition to standard chemotherapy. Even within the subset of patients with FLT3-ITD-positive disease, the addition of sorafenib did not result in an improved EFS or OS, and was instead associated with an increased incidence of adverse events.^80^ Most recently, Rollig *et al.*^81^ investigated the combination of sorafenib with standard chemotherapy in a multicenter randomized controlled phase II trial of 267 patients age 60 or younger with newly diagnosed AML. In this study, patients were randomly assigned to receive two cycles of standard ‘7+3' induction therapy followed by three cycles of high-dose cytarabine as consolidation therapy in combination with either sorafenib (400 mg twice daily) or placebo. Patients assigned to the sorafenib group also received 12 months of sorafenib maintenance therapy after their last consolidation cycle. After 3 years the primary end point, event-free survival, was achieved in 40% of patients in the sorafenib group, versus 20% in the placebo group (with an unadjusted hazard ratio of 0.64).^81^ The occurrence of grades 3–5 diarrhea, rash, fever and bleeding were significantly increased among patients receiving sorafenib (occurring in 11, 7, 54 and 7%, respectively). Importantly, only 17% of the individuals enrolled in this study were positive for the FLT3-ITD mutation. The observed benefit in FLT3-ITD-negative AML may in part be explained by off-target inhibition of other tyrosine kinases, such as c-KIT, PGFR and RAF kinase. Alternatively as suggested by the observed increased in FLT3 receptor ligands level activation of wild-type FLT3 TK may become a driver of leukemogenesis in patients receiving standard chemotherapy.^82^ By targeting FLT3 TK in the period immediately after cytarabine or daunorubicin therapy, sorafenib may exert significant anti-leukemic activity even in FLT3-ITD-negative cells.

In an interesting parallel to the use of TKI in Philadelphia-chromosome-positive leukemias, inhibition of FLT3 TK has also shown a promising role in the post-allo-HSCT setting, either as maintenance therapy or treatment of relapse. This was explored recently by Chen *et al.*^83^ in a phase I trial of 22 patients with FLT3-ITD-positive disease who received sorafenib as maintenance therapy following allo-HSCT. In addition to establishing safety and feasibility, the authors of this study reported rates of PFS and OS which compared favorably to historical controls, particularly in the subset of patients in CR1 or CR2 at the time of transplant. In a retrospective analysis of six patients status post allo-HSCT, sorafenib as maintenance therapy (*n*=5) or treatment of relapse (*n*=1) resulted in a median PFS of 16 months, with all patients remaining in molecular remission (that is, FLT3-ITD-negative by PCR).^84^ Interestingly, skin graft-versus-host-disease occurred shortly after initiation of therapy in five out of these six patients. In addition to its action as a TKI, sorafenib may therefore possess an immunomodulatory role, which synergizes with the graft-versus-leukemia effect.^85^ Two phase I trials of sorafenib use in the peri-transplant setting are currently underway (NCT01398501 and NCT01578109).

#### Midostaurin

Midostaurin is another first-generation FLT3 TKI with significant but transient single-agent activity in patients with AML.^86, 87^ As with sorafenib, its effects are limited by the rapid emergence of resistance. Combinations of midostaurin and existing chemotherapy regimens are currently under investigation: results of a phase I and combined phase I/II trial of midostaurin and azacitidine have recently been published, demonstrating the tolerability and efficacy of this combination in patients with AML. In their cohort of 17 patients with a median age of 73, Cooper *et al.*^88^ established a MTD level of 75 mg PO twice daily with no observed dose-limiting toxicities. In another phase I/II trial of the combination of midostaurin and azacitidine was tested in patients with AML (primary or secondary) and MDS, Strati *et al.* reported a lower MTD of 50 mg PO twice daily. The combination achieved an overall response rate of 26%, with median remission duration of 20 weeks. Although no DLT were observed in this study, neutropenia, thrombocytopenia and anemia developed in 96, 94 and 61% of patients, as well as infections and a decreased left ventricular ejection fraction in 56 and 11% of patients, respectively.^89^ In a recent randomized, double-blind trial of 717 patients with previously untreated FLT3 (ITD and TKD) positive AML, Stone *et al.* explored the role of midostaurin in combination with a standard ‘7+3' induction regimen and high-dose cytarabine consolidation therapy. Patients randomized to the midostaurin treatment arm also received midostaurin as maintenance therapy for one year. Although no difference in the rate of CR was observed between the midostaurin and placebo arms, patients receiving midostaurin had a significantly higher OS and EFS (with a hazard ratio of 0.77 and 0.80, respectively).^90^

#### Quizartinib

Second-generation inhibitors such as quizartinib have been designed to specifically target the FLT3 kinase, in order to reduce toxicity from off-target effects. In addition to this increased selectivity, quizartinib also possesses a good bioavailability and a half-life of more than 24 h, which allows for a more continuous FLT3 inhibition. A phase I study of oral quizartinib in 76 patients with relapsed/refractory AML was able to achieve a hematological response in 30% of patients, and a CR in 13% regardless of FLT3 mutational status. Among patients with FLT3-ITD, the rate of hematologic response increased to 53%, with ~23% of patients achieving CR. Patients were able to tolerate doses of up to 200 mg/day, with grade 3 QT interval prolongation as the only DLT.^91^ In one phase 2 trial, 137 patients with relapsed/refractory AML were given quizartinib monotherapy. The agent was administered in 28-day cycles at doses of 90 mg/day in females and 135 mg/day in males; the most common treatment toxicities were nausea and vomiting in 38 and 26% of patients, anemia in 29%, QT interval prolongation in 26%, febrile neutropenia in 25%, diarrhea in 20% and fatigue in 20%. The rate of composite CR (that is, CR, CR with incomplete platelet recovery and CR with incomplete hematological recovery) and the median OS were 34% and 25.6 months in FLT3-ITD-negative cases, and 44% and 23.1 months in FLT3-ITD positive cases. The observed benefit in FLT3-ITD-negative disease may be explained by off-target effects, or by the upregulation of the FLT3 TK pathway as suggested by the rise in FLT3 receptor ligand levels in patients receiving standard chemotherapy.^92^ Once again however, the response to FLT3 TKI is limited by the rapid emergence of resistance: the median duration of remission was only 5 weeks in patients with FLT3-ITD-positive AML.^93^ Interim analysis of a phase I/II trial of quizartinib in combination with azacitidine or cytarabine (NCT01892371) reported an overall response rate of 82% in FLT3-ITD-positive AML, MDS or chronic myelomonocytic leukemia.^94^ A phase III trial comparing quizartinib monotherapy to salvage chemotherapy in relapsed and refractory AML (NCT02039726) is similarly underway.

#### Crenolanib

Crenolanib besylate is an orally available second-generation FLT3 TKI, with activity against FLT3-ITD and FLT3-TKD mutants. Unlike other FLT3 TKIs, which are subject to the emergence of resistance conferring kinase domain mutations (such as D835Y), crenolanib appears to possess extensive ‘pan-kinase' inhibition of secondary TKD mutations. Using concentrations far below the clinically achievable plasma levels, Smith *et al.* were unable to identify any single TKD mutation able to confer resistance to crenolanib.^95^ In a phase II study of 38 patients with FLT3-mutated AML (including relapsed and refractory patients), crenolanib administered at doses of 200 mg/m^2^ per day three times a day in 28 days cycle achieved a median EFS and OS of 8 and 19 weeks, respectively.^96^ Crenolanib is currently being studied in multiple clinical trials in AML patients, both with and without FLT3-mutated AMLs.

### STAT inhibitors

STAT3 tyrosine phosphorylation is upregulated in up to 50% of AML cases and confers a worse prognosis. Activation of the STAT3 signaling pathway is also stimulated by the FLT3 receptor ligand,^97^ and may represent a key step in the development of FLT3 TKI resistance. Several small molecules of STAT3 inhibitors have been developed and are currently being investigated for the treatment of AML: in 2011, Redell *et al.* showed decreased STAT3 phosphorylation and induction of apoptosis in AML cell lines treated with the STAT3 inhibitor C188-9.^98^ More recently, the optimized compound MM-206 demonstrated a dose-dependent induction of apoptosis in AML cell lines cultured in the presence of bone marrow stromal cells. The anti-tumor activity of MM-206 was confirmed *in vivo* by reducing blast count and improving survival in AML-engrafted mice.^99^ OPB-31121 is a small molecule inhibitor of STAT3 and STAT5 phosphorylation, which has demonstrated activity in advanced solid tumors.^100^ Treatment of various leukemic cell lines with this compound resulted in significant growth inhibition, including FLT3-ITD-positive AML cells.^101^ Importantly, OPB-31121 was able to overcome FLT3 receptor ligand-induced STAT3 phosphorylation, and may help to prevent the emergence of resistance in patients receiving FLT3-ITD TKI. Other STAT3 inhibitors that work as antisense oligonucleotide (ASO) for STAT3 are in clinical trials in hematological malignancies including AML and will have to wait to see their efficacies in near future.

### IDH1/IDH2 small molecule inhibitors

Gain of function mutations in IDH-1 and IDH-2 enzymes are found in approximately 20% of cases.^7^ Recent attempts have been made to target these mutant enzymes as a potential treatment for AML. In 2013, Wang *et al.*^102^ published the results of AGI-6780, a small molecule inhibitor of the R140Q mutant IDH-2 enzyme. In an *ex vivo* model of primary human AML cells, treatment with AGI-6780 was able to overcome the differentiation block of leukemic cells. Recently, the IDH-2 inhibitor AG-221 was found to confer a dose-dependent survival benefits in a primary human IDH-2 mutant AML xenograft model. At a cellular level, AG-221 treatment was associated with an initial phase of CD45+ blast cells proliferation, followed by cellular differentiation.^103^ A phase I trial of AG-221 in IDH-2 mutant leukemia is currently underway (NCT01915498). IDH-1 mutant enzymes are also the targets of new therapeutic inhibitors: Preliminary results of a phase I trial of the small molecule inhibitor AG-120 (NCT02074839) demonstrated hematological response in 7 out of 14 IDH-1 positive patients, including 4 CR.^104^

### Clofarabine

Clofarabine is a second-generation purine nucleoside analog approved for the treatment of relapsed or refractory pediatric acute lymphocytic leukemia. In AML, it has shown activity and tolerability as a single-agent, administered intravenously at doses of 20–30 mg/m^2^ for 5 days, with overall response rates of ~40%.^105, 106^ In a 2008 randomized study of 70 patients aged 60 years and older, it was reported a CR rate of 63% using the combination of clofarabine with low-dose cytarabine, compared with 31% with clofarabine alone.^107^ In a study of 320 patients over the age of 55 with relapsed/refractory AML, the combination of clofarabine and cytarabine achieved significantly higher rates of CR, CR with incomplete platelet count and DFS when compared with cytarabine alone.^108^ Although neither of these studies was able to show an improved OS, these results suggest a synergistic action between clofarabine and cytarabine, and have spurred interest in the combination of these two agents. Recently a study was published showing the results of a phase 2 trial of clofarabine and low-dose cytarabine in older patients with newly diagnosed AML.^109^ In this study, 118 patients age 60 or older (median age of 68 years) received induction therapy with clofarabine at 20 mg/m^2^ on day 1 through 5 and low-dose cytarabine at 20 mg subcutaneously twice daily on day 1 through 10. In an attempt to improve survival, this was followed by consolidation and maintenance therapy with up to 18 cycles of clofarabine and low-dose cytarabine alternating with decitabine. CR was achieved in 60% of cases, with a median OS of 11.1 months, and a median RFS of 14.1 months. The regimen was well-tolerated, with a 4-week mortality rate of 3%. The most common non-hematological toxicities reported in this study included nausea in 81% of cases, elevated liver transaminases and bilirubin in 64 and 47% of cases, as well as rash in 56% of cases. Clofarabine may thus represent a well-tolerated addition to low-dose cytarabine in elderly patients unable to receive standard chemotherapy or allo-HSCT for consolidation therapy. Clofarabine has shown similarly promising results in younger patients when combined with the standard ‘7+3' induction regimen. In a study of 57 newly diagnosed patients under the age of 60, the combination of clofarabine (at 22.5 mg/m^2^ IV daily for 5 days) with idarubicin and cytarabine used as a frontline induction and consolidation therapy achieved CR rates of 74% and a median EFS of 13.5 months (the median OS and RFS had not been reached by a median follow-up of 10.9 months).^110^ A phase I/II study of frontline clofarabine, cytarabine and idarubicin in patients with intermediate and poor risk AML is currently underway (NCT00838240). The potential role of clofarabine in the peri-transplant setting is also currently under investigation. In a multicenter two-stage phase II trial, 84 patients with relapsed and refractory AML received clofarabine (at 30 mg/m^2^ for 5 days) in combination with cytarabine as salvage therapy, followed by 4 days of clofarabine and one dose of melphalan in chemo-responsive patients with HLA-compatible donors. Out of the 56 patients who underwent allo-HSCT, CR was achieved in 50 (including 11 CR with incomplete platelet recovery and 10 CR by chimerism). The 2-year OS of 43% compared favorably with historical controls.^111^ Although randomized controlled trials are needed to clearly delineate the role of clofarabine in AML these studies present promising results in support of this agent, particularly in combination with cytarabine in the elderly.

In addition to its activity as an intravenous agent, clofarabine has also captured interest as an oral agent for the treatment of AML. Unlike previous purine nucleoside analogs, clofarabine is able to resist acidic pH as well as phosphorolytic cleavage by gastrointestinal *Escherichia coli*. As a result it possesses a bioavailability of roughly 50%.^112^ In a phase I/II study of 35 patients 60 years or older with relapsed/refractory AML or high-risk MDS the feasibility and efficacy of oral clofarabine combined with low-dose cytarabine was investigated as a first-line therapy.^113^ At a MTD of oral clofarabine of 20 mg per day for 5 days, CR was achieved in 42% of patients (including CR with incomplete count recovery in 4%). Most importantly, more than 50% of cycles administered at the MTD were given in the outpatient setting. In a population at an increased risk of TRM, oral clofarabine may thus offer a valuable addition to reduced intensity chemotherapy.

### Monoclonal antibodies

Monoclonal antibodies exert their anti-tumor activity through direct antibody-dependent cytotoxicity or through the conjugation of cytotoxic agents, which allows for the targeted delivery of potent chemotherapy to neoplastic cells. Gemtuzumab ozogamicin (GO) is a humanized recombinant antibody directed at CD33, a transmembrane protein expressed on cells of myeloid lineage. The antibody is conjugated to the DNA-cleaving cytotoxic agent calicheamicin, and is internalized by CD33-positive cells. GO received FDA approval in 2000 for the treatment of CD33-positive AML in patients 60 years or older at first relapse. However in 2009, interim analysis of a randomized clinical trial of 637 patients with newly diagnosed AML revealed increased fatal toxicity with no improvement in CR, disease-free survival or OS in patients receiving GO in combination with standard chemotherapy.^114^ The trial was prematurely terminated and FDA approval was rescinded. Despite its removal from the market, a 2014 meta-analysis of five randomized clinical trials demonstrated a decrease in relapse and improved survival in patients receiving GO in addition to standard chemotherapy.^115^ A subset analysis further revealed that this survival benefit was limited to patients with a favorable or intermediate cytogenetic-risk profile. Most recently, a randomized trial of 237 patients 60 years of age or older ineligible for intensive chemotherapy showed an improved OS (hazard ratio of 0.69) in those assigned to GO induction and consolidation compared with best supportive care.^116^ This survival benefit was again most pronounced in patients with an intermediate to favorable cytogenetic-risk profile. Although further studies are required to fully delineate the effects of gemtuzumab in the treatment of AML, these data suggest a benefit among elderly patients with favorable or intermediate cytogenetic-risk profile. CD37 is a transmembrane protein that is expressed in high levels on maturing B cells. Although its exact function has not yet been elucidated, it is upregulated in non-Hogkin lymphoma and chronic lymphocytic leukemia.^117^ Initially conceived as a therapy for B-cell malignancies, AGS67E is a fully human anti-CD37 IgG antibody that is conjugated with monomethyl auristatin E (MMAE), a potent microtubule-disrupting agent. AGS67E allows for the selective delivery of MMAE to CD37-positive malignant cells and results in apoptosis. Although CD37 is minimally expressed on normal myeloid stem cells it was recently demonstrated to have differential expression of CD37 on the surface of CD34^+^/CD38^−^ AML stem cells. *In vitro* treatment of leukemic cells with nanomolar concentrations of AGS67E resulted in cytotoxicity, altered cell growth and apoptosis in seven out of 16 AML cell lines. The administration of AGS67E was further found to significantly decreased tumor engraftment in a murine xenograft model of AML, resulting in undetectable leukemic cell levels in three out of four AML samples. CD37 may thus represent a novel therapeutic target for the selective inhibition of leukemic cell growth. Although still at an early stage of its clinical development, AGS67E is a promising new therapy. More importantly, the identification of unique molecular markers expressed on the surface of leukemic cells is an emerging avenue for the discovery of novel targeted therapies against AML.

### CART therapy

Chimeric antigen receptors are synthetic T-cell receptors with antibody-like specificity. They combine a single-chain variable fragment from a monoclonal antibody with the transmembrane and intracellular domains of a T-cell receptor. This allows for the creation of a host-derived population of chimeric antigen receptor-T (CART) cells, which can be directed at a pre-determined antigen. CD19-directed CART cell therapy has shown exciting efficacy in the treatment of acute lymphoblastic leukemia and B-cell lymphoma. Although this treatment does not distinguish between malignant and healthy CD19 cells, patients tolerate the depletion of CD19 lymphocytes with relatively little morbidity. In contrast to this, depletion of the normal cells of myeloid lineage is associated with unacceptable neutropenia, and attempts to apply CART cell therapy to the treatment of AML have had to focus on identifying an appropriate antigen to target malignant cells while sparing non-malignant myeloid cells. Kenderian *et al.* recently developed a CD33-specific CAR based on the single-chain variable fragment of gemtuzumab ozogamicin. They have reported potent *in vitro* activity of such CD33-specific CART cells against AML cell lines.^118^ CD33-specific CART therapy also prolonged survival in AML xenografts. However CD33 is expressed on normal cells of myeloid lineage and the reported anti-tumor effects were associated with profound cytopenias. Using electroporation of CD33-specific CAR mRNA into human T cells, the authors are able to induce the transient expression of anti-CD33 CAR,^118^ which may confer clinically significant anti-tumor activity while avoiding long-term myelosuppression. In an attempt to selectively target leukemic myeloid cells, others have focused instead on the β member of the folate receptor family (FRβ). This receptor subtype is primarily expressed on myeloid-lineage hematopoietic cells, and is upregulated in the setting of malignancy.^119^ FRβ is expressed in 70% of cases of primary AML, and its expression can be further upregulated following treatment with all-trans retinoic acid.^120^ In a recent publication the effect of FRβ-specific CART cells therapy *in vitro* as well as in AML xenograft was reported. The study demonstrated lytic activity against FRβ positive AML cell lines both *in vitro* and *in vivo*. Importantly, no evidence of toxic activity against healthy human CD34-positive stem cells was observed *in vitro*.^121^ Although still in its early stages, CART cell therapy may thus provide an alternative mechanism of treatment for patients with relapsed/refractory AML.

## Conclusion

AML is a biologically and clinically heterogeneous disease. Although advances in supportive care and prognostic risk stratification have optimized established therapies, overall long-term survival remains poor. Elderly patients who account for the majority of newly diagnosed cases are more likely to present with an adverse cytogenetic profile. At the same time the increased risk of TRM often precludes this population of patients from receiving optimal chemotherapy or stem cell transplantation. Novel targeted therapies offer the promise of effective anti-leukemic activity with reduced toxicity from off-target effects. However given the molecular diversity of AML, it is unlikely that targeted therapies such as FLT3 tyrosine kinase inhibitors will provide a single ‘magic bullet' against this disease. Rather the development of new treatments, in concert with improved genetic profiling and risk stratification, can be expected to result in incremental gains in remission and survival. Furthermore in addition to mutated enzymes and upregulated pathways, the identification of unique cell surface markers can provide a therapeutic target for recombinant monoclonal antibodies or chimeric antigen receptors. Here, the challenge lays in selectively targeting leukemic myeloid cells while sparing non-malignant myeloid precursors. Lastly, the development of well-tolerated oral therapies, such as clofarabine, will increasingly broaden the range of available treatment for elderly patients at a higher risk of mortality from standard chemotherapy regimens. We are looking to a new era in the treatment of AML to begin with novel agents so we can achieve better responses with prolong OS particularly for patients with relapsed or refractory diseases and poor cytogenetic features.

## Figures and Tables

**Table 1:** WHO classification of AML and related neoplasms

*Types*	*Genetic abnormalites*
AML with recurrent genetic abnormalities	AML with t(8:21)(q22;q22); RUNX1-RUNX1T1
	AML with inv(16)(p13.1q22) or t(16;16)(p13.1;q22); CBFB-MYH11
	APL with PML-RARA
	AML with t(9;11)(p21.3;q23.3); MLLT3-KMT2A
	ML with t(6;9)(p23;q34.1); DEK-NUP214
	AML with inv(3)(q21.3q26.2) or t(3;3)(q21.3;q26.2); GATA2, MECOM
	AML (megakaryoblastic) with t(1;22)(p13.3;q13.3); RBM15-MKL1
	AML with BCR-ABL1 (provisional entity)
	AML with mutated NPM1
	AML with biallelic mutations of CEBPA
	AML with mutated RUNX1 (provisional entity)
AML with myelodysplasia-related changes	
Therapy-related myeloid neoplasms	
	AML with minimal differentiation
	AML without maturation
	AML with maturation
	Acute myelomonocytic leukemia
	Acute monoblastic/monocytic leukemia
	Acute erythroid leukemia
	Pure erythroid leukemia
	Acute megakaryoblastic leukemia
	Acute basophilic leukemia
	Acute panmyelosis with myelofibrosis
Myeloid sarcoma	
Myeloid proliferations related to Down syndrome	Transient abnormal myelopoiesis
	ML associated with Down syndrome

Abbreviations: AML, acute myeloid leukemia; APL, acute promyelocytic leukemia; ML, myeloid leukemia; WHO, World Health Organization.

**Table 2:** Prognostic-risk group based on cytogenetic and molecular profile

*Prognostic-risk group*	*Cytogenetic profile alone*	*Cytogenetic profile and molecular abnormalities*
Favorable	t(8:21)(q22;q22)	t(8:21)(q22;q22) with no c-KIT mutation
	inv(16)(p13;1q22)	inv(16)(p13;1q22)
	t(15;17)(q22;q12)	t(15;17)(q22;q12)
		Mutated NPM1 without FLT3-ITD (CN-AML)
		Mutated biallelic CEBPA (CN-AML)
Intermediate	CN-AML	t(8:21)(q22;q22) with mutated c-KIT
	t(9;11)(p22;q23)	CN-AML other than those included in the favorable or adverse prognostic group
	Cytogenetic abnormalities not included in the favorable or adverse prognostic risk groups	t(9;11)(p22;q23)
		Cytogenetic abnormalities not included in the favorable or adverse prognostic risk groups
Adverse	inv(3)(q21q26.2)	TP53 mutation, regardless of cytogenetic profile
	t(6;9)(p23;q34)	CN with FLT3-ITD
	11q abnormalities other than t(9;11)	CN with DNMT3A
	−5 or del(5q)	CN with KMT2A-PTD
	−7	inv(3)(q21q26.2)
	Complex karyotype	t(6;9)(p23;q34)
		11q abnormalities other than t(9;11)
		−5 or del(5q)
		−7
		Complex karyotype

Abbreviations: AML, acute myeloid leukemia; ITD, internal tandem duplications.
